# Oral disorders in Children with Prader–Willi syndrome: a case control study

**DOI:** 10.1186/s13023-020-01566-7

**Published:** 2021-01-06

**Authors:** Sonal Lavakumar Budihal, Aya Mohammad Ahmad, Adama Sani Usman, Anusha Sreejith, Jayadevan Sreedharan

**Affiliations:** Gulf Medical University, Ajman, United Arab Emirates

Dear Editor,

This letter is about the article published by Carla Munné-Miralvés et al. [1] about the oral disorders in children with Prader–Willi syndrome. The objective of the study was to evaluate the oral conditions of children with Prader–Willi syndrome and to establish a prevention protocol. We acknowledge the authors’ efforts for choosing this topic for their research. We would like to raise some points regarding the objectives, methodology and results in the study.

It is commendable that the authors could find 30 cases of PWS in the Barcelona area alone for such a rare syndrome, as the prevalence reported was only 3.1/100,000 live births in Europe (mentioned in the article). Moreover, the authors have not given any justification regarding the sample size calculation.

In the methodology, the authors mentioned that the design used is a ‘cross-sectional observational case–control study’, and in the title it is a case–control study. A case–control study is a retrospective study to establish the association between a disease and related factors, and there is no such study design called “cross-sectional observational case–control study”. Since this study did not investigate any factors causing PWS, case–control study design is not an appropriate study design in this situation.

Statistically significant difference was observed with respect to three oral parameters; stimulated saliva in ml/min, (0.475 in PWS group and 0.848 in the control group), pH (6.15 in PWS group and 7.53 in control group) and DMFT (2.5 in the PWS group and 0.93 in the control group) Table 2 in the article. As per the literature, saliva is slightly acidic with pH ranges from 6 to 7 [2]. But the results from this study showed that pH value is abnormal for control group, which is unlikely. Also, regarding stimulated flow rate of saliva, literature shows a normal range of 4–5 mL/min [3]. As per the current study, the stimulated saliva rate was very low in both groups and showed a statistical significance. DMFT is not a continuous variable. Therefore, calculation of mean and standard deviation, and t-test for testing the hypothesis are inappropriate in this context. Authors may clarify these points.

The results for Chi square test was not shown anywhere in the article, but it was mentioned in the methodology. Though p-value showed statistically significant, the correlation between both variables is weak, which in turns becomes insignificant. Lastly, no preventive protocol was established in this study for PWS assessment as it was one of the objectives of the study. Also, the conclusion did not fulfill the objectives of this study.

We really appreciate the authors for their initiative, but inappropriate methodology and selection of statistical methods lead to fallacious results.

## Author’s response

We thank the writers of the letter for their interest in our work, and also thank the editors for the opportunity to respond to the comments and criticisms made and thus to extend the discussion of our study. First, we stress that Sant Joan de Deú University Hospital is a referral institution for paediatric rare diseases. It is the only one in Spain with a paediatric dentistry service, with a career stretching back more than 50 years. This unusual situation enabled us to recruit a large sample (n = 30) of patients diagnosed with PWS. Second, in the comparison of the DMFT index in the two groups, we used the mean values of this variable and then applied the Student’s “t” test. We then categorized this variable as discrete and after applying the chi squared test we obtained the same significant differences between the two groups. This information did not appear in the body of the text so as not to duplicate the data presented in the tables. Thirdly, with regard to the comment about our results, we stress that the salivary values were double checked. Although in the control group these values differed slightly from the standard parameters, the important point is that with respect to the PWS group the differences were significant. Finally, we took full account of the comments of the three reviewers during the submission process and introduced all the changes suggested. Nevertheless, we agree that the term “cross-sectional study” would have been more appropriate in the title.

Yours sincerely,

Lluís Brunet-Llobet, MD, DDS, PhD

Corresponding author

## Data Availability

Not applicable.
